# Prenatal diagnosis of Sex determining region Y -box transcription factor 2 anophthalmia syndrome caused by germline mosaicism using next-generation sequencing: A case report

**DOI:** 10.18502/ijrm.v21i8.14022

**Published:** 2023-09-20

**Authors:** Pooneh Nikuei, Ph.D., Zahra Khashavy, Mohammad Ali Farazi Fard, Shahrzad Tabasi, Ari Zeidi5 B.Sc. Student, Parnian Pourkashani, Zahra Tabatabaei, Ebrahim Eftekhar, Mozhgan Saberi, Frouzandeh Mahjoubi

**Affiliations:** ^1^Hormozgan State of Welfare Organization, Bandar Abbas, Iran.; ^2^Gambron Royan Infertility Center, Bandar Abbas, Iran.; ^3^Persian Bayan Gene Research and Training Center, Shiraz, Iran.; ^4^Department of Clinical Pharmacy, Faculty of Pharmacy, Hormozgan University of Medical Sciences, Bandar Abbas, Iran.; ^5^New York University, NY, USA.; ^6^Iran University of Medical Sciences, Tehran, Iran.; ^7^Molecular Medicine Research Center, Hormozgan Health Institute, Hormozgan University of Medical Sciences, Bandar Abbas, Iran.; ^8^Department of Medical Genetics, Institute of Medical Biotechnology, National Institute of Genetic Engineering and Biotechnology, Tehran, Iran.

**Keywords:** Anophthalmos, SOX2 anophthalmia syndrome, Mosaicism.

## Abstract

**Background:**

Sex determining region Y box transcription factor 2 (SOX2) mutations lead to bilateral anophthalmia with autosomal dominant human inheritance. SOX2 mutations could result in severe ocular phenotypes usually associated with variable systemic defects. Most patients described with SOX2 anophthalmia syndrome possessed de novo mutations in this gene.

**Case Presentation:**

In this case report, we describe 2 brothers with mental retardation and bilateral anophthalmia caused due to SOX2 germline mosaicism in unaffected parents. Next-generation DNA sequencing was carried out to determine the family's possible cause of genetic mutation. Sanger sequencing was performed on the patients and their parents. Prenatal diagnosis was done in both pregnancies of the older brother's wife via chorionic villus sampling. A novel heterozygous pathogenic frameshift deletion variant (exon1:c.58_80del:p.G20fs) was identified in the *SOX2* gene, which was confirmed by Sanger sequencing in both affected brothers and did not exist in healthy parents, indicating germline mosaicism.

**Conclusion:**

Most SOX2 mutations known look to arise de novo in probands and are diagnosed through anophthalmia or microphthalmia. Prenatal diagnosis should be offered to healthy parents with a child with SOX2 mutation every pregnancy.

## 1. Introduction

Mutations identified in the offspring but absent in the parents are often known as de novo mutations. There are 2 scenarios regarding de novo mutations; it either arose as somatic mutations during embryonic development or occurs in one of the parents gametes (germline or gonadal mosaicism). When 2 or more children are affected from unaffected parents the likelihood of gonadal mosaicism is the most probable explanation (1, 2). Different types of mosaicisms show which organs of the body manifest the mutated cells and their ability to pass on to progeny. “These consist of isolated germline mosaicism, somatic mosaicism, and integration of germline and somatic mosaicism" (3, 4). Genetic mutations that occur due to neurological, neuropsychiatric, and neurodevelopmental disorders are usually considered as either inherited or de novo germline mutations (5).

SRY (sex determining region Y) -box transcription factor 2 (SOX2) variants and deletions are known causes of anophthalmia and microphthalmia (6). SOX2 mutations lead to bilateral anophthalmia with an autosomal dominant pattern in humans. Anophthalmia is a congenital absence of the eyes which leads to congenital blindness (7). Extraocular symptoms are brain malformations, male genital tract defects, postnatal growth retardation, facial dysmorphism, seizures, and learning difficulty (8).

De novo mutations in *SOX2* gene are responsible in most cases with SOX2 anophthalmia syndrome.

Most patients affected to this syndrome have bilateral anophthalmia or microphthalmia as the main phenotype. “SOX2 mutations can be presented with anophthalmia esophageal-genital syndrome (OMIM 600992), an association of anophthalmia or microphthalmia, esophageal atresia with or without tracheoesophageal fistula, and urogenital anomalies, usually cryptorchidism, hypospadias, and micropenis" (9).

In today's world, the next-generation sequencing technique in parent-offspring can be utilized directly to study the frequency of all de novo mutations in the entire genome. We reported a familial recurrence of a variable clinical presentation from anophthalmia and hydrocephaly to the absence of ocular involvement in 2 affected Iranian brothers. Both had a heterozygous mutation in the *SOX2* gene due to germinal mosaicism. The results of prenatal diagnosis showed affected fetuses with the same mutation in the first and second pregnancies of the older patient's wife. One of the fetuses was therapeutically aborted, and in another pregnancy, the mother refused to terminate her pregnancy despite the affected fetus being diagnosed with prenatal diagnosis. The disease phenotypes were anophthalmia and hydrocephaly in a newborn girl.

## 2. Case Presentation

The case was a 28-yr-old man affected by mild mental retardation who was working as a car repairman and married to a non-consanguineous healthy woman. He had normal eye structures and vision. They visited for genetic counseling before pregnancy.

**Figure 1:**
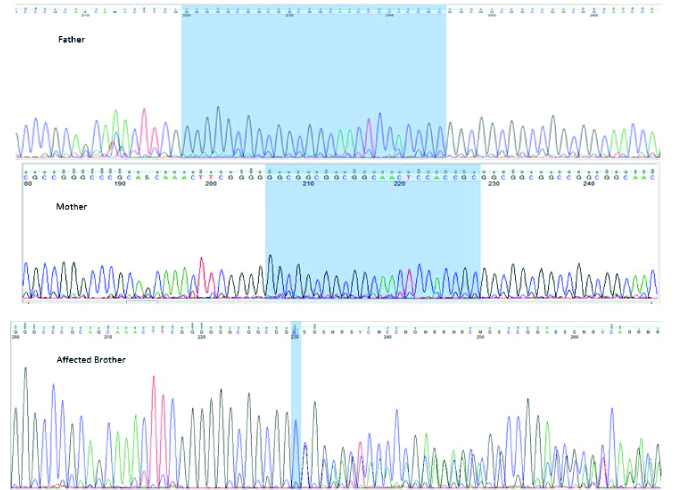
Pedigree of the family. Proband is shown with a black arrow.

His brother, a 17-yr-old boy, was affected by severe mental retardation and anophthalmia. Their parents were healthy with non-consanguineous marriage (Figure 1 pedigree). Both brothers had a normal karyotype.

For identification of the possible genetic cause whole exome sequencing (WES), was applied. DNA was extracted from the peripheral blood.

The results were subsequently analyzed with a Burrows-Wheeler aligner (10), Genome Analysis Toolkit (11), and ANNOVAR (12). Population databases (such as ExAC browser, gnomAD, and Kaviar VARiants) and local database used for filtering. The mean coverage was 100X.

Sanger sequencing was performed on the patient's brothers (both affected and healthy) and also his parents. Additionally, the patient's father's semen sample was assessed by Sanger sequencing to consider germline mosaicism occurrence. Regarding the patient's wife's pregnancy, prenatal diagnosis was completed in both pregnancies via chorionic villus sampling.

**Figure 2:**
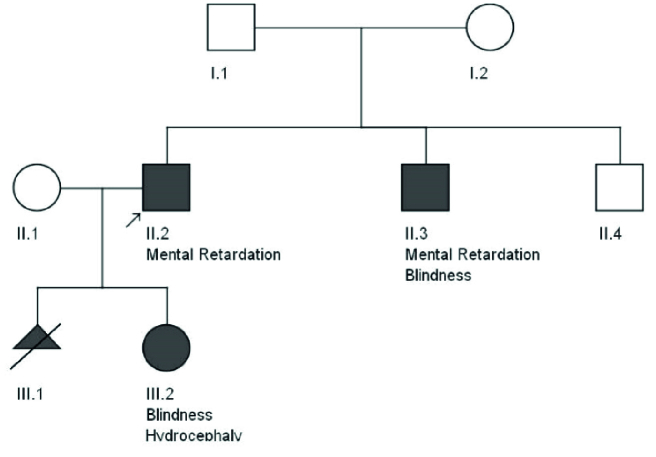
Sanger sequencing results of the family.

Results of WES revealed a novel heterozygous frameshift deletion mutation in the SOX2 (NM_003106:exon1:c.58_80del:p.G20fs) in both affected brothers. To confirm the found variant, Sanger sequencing was performed. Peripheral blood samples from parents and healthy siblings were taken. Sanger sequencing confirmed the mutation in the heterozygous state in both affected brothers with the mild and severe phenotype (Figure 2). However, this mutation was not found in their parents, indicating germline mosaicism. Based on the American College of Medical Genetics and Genomics guidelines, the identified mutation is classified as a pathogenic mutation. Also, the patient's wife and his healthy brother did not have the identified mutation. Results of Sanger sequencing on the patient's father's semen did not show the mutation.

After genetic counseling and pedigree drawing (Figure 1), based on the results of their tests, autosomal dominant inheritance of this mutation and 50% recurrence risk was explained to the older brother's wife. Preimplantation genetic diagnosis and Prenatal diagnosis were offered for pregnancies. The family decided to do PND in each pregnancy. In the first pregnancy, PND was carried out and the results revealed that the fetus was a heterozygote for the identified mutation in the exon 1: c.58_80del in the *SOX2* gene, therefore, was affected. As a result, the mother decided to terminate her pregnancy. In the next pregnancy, PND results again showed heterozygote mutation. Still, the mother decided to keep the fetus despite awareness of the fetus's involvement with severe hydrocephaly detected on fetal ultrasound in the 18 ${}^{\text{th}}$ wk of pregnancy. The result of the pregnancy was a girl with anophthalmus and hydrocephaly. Both orbital cavities were severely hypoplastic with abundant glial tissue. Brain magnetic resonance imaging revealed a significant dilation of the lateral and third ventricles and hypoplastic corpus callosum.

### Ethical considerations

The study was approved by the Ethics Committee of Hormozgan University of Medical Sciences, Bandar Abbas, Iran (Code: IR.HUMS.REC.1398.458). Oral consent was obtained from parents.

## 3. Discussion

In this case, a novel heterozygote frameshift deletion mutation was found in the *SOX2* gene inherited as germline mosaicism. “Mutations in this gene are shown to cause microphthalmia, syndromic 3, autosomal dominant, optic nerve hypoplasia, and abnormalities of the central nervous system" (OMIM # 206900).

SOX2 anophthalmia syndrome is a clinically identified disorder found in 15-20% of patients with bilateral anophthalmia (8, 13). Extra-ocular anomalies are usual in affected persons. The variable phenotypic expression has been announced for SOX2 mutations (8). The frequent SOX2-associated ocular phenotype is bilateral clinical anophthalmia (14).

We reported here a patient (the older brother) who had a pathogenic heterozygous mutation in the *SOX2* gene with no detectable eye malformation, having a few reports previously (15). Despite being the first patient of the family having SOX2 mutation, he had normal eyes that support the hypothesis of SOX2 involvement in different developmental pathways.

Based on the tissue, and timing of the *SOX2* gene expression, plus the effect of other transcription factors, variable features are expected. For example, SOX2 paralogues of the B1 SOX group, such as SOX1 or SOX3, have been shown that are able to cover up SOX2 deficiency in animal models (8).

The hypothetical protein product is supposed to cause haploinsufficient SOX2 function (8). Brain malformations are one of the features in SOX2 anophthalmia syndrome often found as mesial temporal malformations, while hydrocephalus is less frequent characteristic (8).

It seems that the SOX2 mutation is causative for hydrocephaly in the affected newborn. Zenteno et al. described monozygotic twin males with c.70 del20 mutation in SOX2. “One of the twins had unilateral anophthalmia and esophageal atresia while the other had normal-sized corneas but a short palpebral fissure on one eye and esophageal atresia" (16).

Chassaing et al. reported a woman with germinal mosaicism (c.70_86del in SOX2), whose 2 affected girls had variable clinical presentations of the SOX2 phenotype. The first child had type III esophageal atresia and a diagnosis of AEG syndrome (Anophthalmia, Esophageal atresia, Genital abnormalities). Interestingly, ultrasound in mothers next pregnancy revealed a fetus progressive hydrocephalus. An autopsy of the female fetus showed a female fetus with normal eyes but brain anomalies and 11 pairs of ribs (8).

Most SOX2 mutations known, look to arise de novo in probands discovered through anophthalmia or microphthalmia (17). Genetic types of anophthalmia can have different mode of inheritance such as autosomal recessive, autosomal dominant, or X-linked inheritance (18). Genetic counseling in anophthalmia is challenging because of the great heterogeneity in anophthalmia etiology.

New progress in recognizing genes responsible for anophthalmia shown by WES can be useful for genetic counseling in patients. Our report documents some of the rare examples of recurrence in SOX2 anophthalmia syndrome. This report shows the significance of mutation analysis of SOX2 in all patients with anophthalmia and/or microphthalmia with neurological features in the association. In the process of genetic counseling for autosomal dominant disorders, somatic mosaicism is well known, and recurrence risk for “new dominant” mutations is often 1-6% (19).

The distinguishing of a de novo mutation as the cause of an autosomal dominant disease in a child had a low recurrence risk in another sibling, and is positive news for parents because it is estimated the same as the normal population. However, PND should be done in each pregnancy, when 2 or more affected children are born to apparently unaffected parents, proposing a recurrence risk of germline mosaicism. This issue will frequently be missed in first-generation offspring when only one child has been affected (20).

Difficult confirmation of mosaicism in germ cell compartments was a limitation of this study. When target tissue is not blood and skin, 2 widely used tissue samples, identification of tissue-limited mosaicism is not easy. “Pasmant suggests that fathers' sperm can be employed for genetic analysis for screening germ line mutations in cases with apparently de novo mutations" (15). In our study, semen sample was taken from patients' fathers to investigate identified pathogenic mutation using Sanger sequencing technique, which showed no mutation. Maternal germline mosaicism cannot be studied since eggs are not easily obtained (2). Genetic counseling and identifying recurrence risks for parents with possible germline mosaicism is recommended.

## 4. Conclusion 

The results of our study suggested that genetic testing of parents is indicated when a SOX2 mutation is identified in a proband and if 2 or more children were affected from unaffected parents germline mosaicism should be considered. We suggest that prenatal diagnosis should be offered in each pregnancy to healthy parents having an affected child with a SOX2 mutation.

##  Conflict of Interest

The authors declare that they have no competing interest.
